# Something to talk about; crosstalk disruption at the neurovascular unit during HIV infection of the CNS

**DOI:** 10.1515/nipt-2024-0003

**Published:** 2024-07-24

**Authors:** Kalpani N. Udeni Galpayage Dona, Mohammed M. Benmassaoud, Cassandra D. Gipson, Jay P. McLaughlin, Servio H. Ramirez, Allison M. Andrews

**Affiliations:** Department of Pathology, Immunology and Laboratory Medicine, College of Medicine, University of Florida, Gainesville, FL, USA; Department of Pharmacology and Nutritional Sciences, University of Kentucky, Lexington, KY, USA; Department of Pharmacodynamics, College of Pharmacy, University of Florida, Gainesville, FL, USA

**Keywords:** neurovascular unit, blood-brain barrier, intercellular, endothelial cells

## Abstract

Although treatable with antiretroviral therapy, HIV infection persists in people living with HIV (PLWH). It is well known that the HIV virus finds refuge in places for which antiretroviral medications do not reach therapeutic levels, mainly the CNS. It is clear that as PLWH age, the likelihood of developing HIV-associated neurological deficits increases. At the biochemical level neurological dysfunction is the manifestation of altered cellular function and ineffective intercellular communication. In this review, we examine how intercellular signaling in the brain is disrupted in the context of HIV. Specifically, the concept of how the blood-brain barrier can be a convergence point for crosstalk, is explored. Crosstalk between the cells of the neurovascular unit (NVU) (endothelium, pericytes, astrocytes, microglia and neurons) is critical for maintaining proper brain function. In fact, the NVU allows for rapid matching of neuronal metabolic needs, regulation of blood-brain barrier (BBB) dynamics for nutrient transport and changes to the level of immunosurveillance. This review invites the reader to conceptually consider the BBB as a router or convergence point for NVU crosstalk, to facilitate a better understanding of the intricate signaling events that underpin the function of the NVU during HIV associated neuropathology.

## Introduction

The notion that the central nervous system (CNS) is an immunologically privileged site has greatly been revised in recent years. Once considered a walled-off or an immunologically inert organ, it is now known that the neurovascular unit (NVU) tightly regulates immune infiltration and immunosurveillance of the brain [1]. This aspect of CNS homeostasis is largely attributed to the role of the blood-brain barrier (BBB) which also controls molecular traffic in and out of the CNS parenchyma [2]. Over the decades, the study of neuroHIV has provided key insights into how the status of the BBB is affected during neuroinflammation. In this review, we use neuroHIV as a case study for examining signal convergence and altered function at the BBB. Although, a compromised BBB is observed in nearly all aspects in which a condition, disease or disorder impairs neurological performance [3], HIV offers a unique perspective into the pathophysiology of the BBB.

The BBB is a unique cellular interface formed by endothelial cells (ECs) that line the walls of cerebral blood vessels. This highly specialized endothelium differs from that seen in most of the vasculature outside of the CNS [4]. Brain ECs are considered barrier forming due to the presence of dense intercellular tight junction complexes that essentially seals off the paracellular route movement of blood solutes. Charged ions, amino acids, glucose, small peptides, non-polar and lipid soluble solutes etc., are allowed to cross the BBB via intricate and highly elaborate intracellular transport mechanisms [5]. The BBB doesn’t just involve one-way transport but it is also specialized in bidirectional movement of metabolites in and out of the brain. Importantly, by virtue of the tight junctions, the BBB closes off or significantly limits immune crossing into the parenchyma. BBB properties can mainly be generated by mature and post-mitotic brain ECs, however the modulation of function and its dynamic changes are governed by regulatory signals from the other members of the NVU [6]. ECs are part of the NVU which also includes astrocytes, pericytes, neurons and microglia which are involved in all aspects of BBB functionality [7]. In a broader sense the NVU allows for rapid adjustments in localized and regional metabolic demand by its regulation of cerebral blood flow which is fundamental for maintaining and providing optimal neurological performance [8].

Consequently, the combined effects of HIV infection can be described as altering the NVU in distinct ways via convergence of crosstalk signaling at the BBB interface. This review will focus on examining crosstalk signaling event between the brain endothelium and the members of the NVU rather than crosstalk in which brain ECs are not directly involved.

## Immune and endothelial crosstalk

In the context of HIV infection, clinical evidence shows 20–35 % of people living with HIV (PLWH) with or without ART had increased BBB permeability as calculated by the ratio of cerebrospinal fluid (CSF) albumin (mg//L) to serum albumin (mg/L) levels [9, 10]. However, the nucleating cause of the BBB hyperpermeability remains incompletely resolved. Prior to the availability and widespread use of ART, unchecked replication of the virus in the brain placed individuals at a high risk for HIV encephalitis and significant neurological deficits [11]. These severe pathologies would create a condition that completely collapsed the normal regulatory mechanisms at the BBB. For example, massive infiltration of immune cells, activated astrocytes and microglia, dysfunction in neurovascular coupling and edema formation are evidence of a critically compromised BBB. Although severe cases of HIV neuroinflammation do occur, ART intervention has dramatically reduced severity to a now more-mild form under the designation of HIV-associated neurocognitive disorder (HAND) with a prevalence of 20–50 % [12]. The persistence of neurocognitive changes is attributed to unresolved BBB dysfunction, neuroinflammation and low levels of viral replication or viral protein production [13].

A key event during HIV pathology is the infiltration of infected monocytes into the CNS via crossing of the BBB and continuing this process throughout the course of infection [14]. CD16+ monocytes develop into a growing immune cell population during HIV infection [15, 16]. Hence, CD16+ serve as a continuous means of trafficking HIV from the periphery into the brain [17, 18]. Moreover, infected monocytes from HIV (such as HIV-1_ADA_) cross the BBB more readily compared to the uninfected monocytes [19]. Although monocytes are not very refractory to replication, differentiated macrophages in the brain are much more susceptible to HIV infection [20, 21]. The infected cells then release virus that proceeds to infect microglia [22].

**Figure 1:**
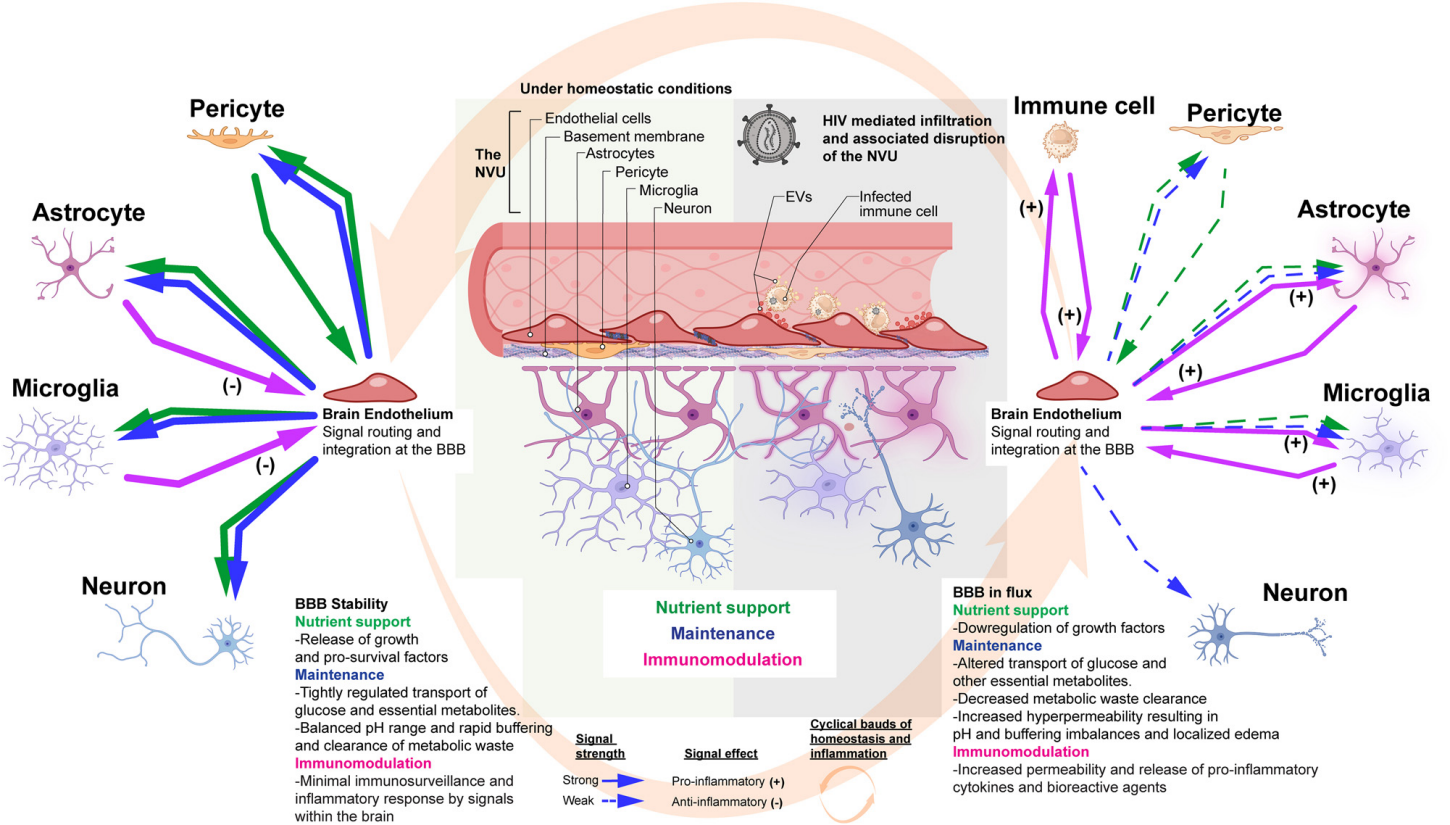
The NVU under homeostasis and neuroinflammation showing crosstalk convergence at the brain endothelium. The brain endothelium forms the BBB, which at the cellular level regulates blood solute entry into the brain parenchyma. The BBB also significantly mediates the clearance of metabolic waste generated within the CNS parenchyma. The schematic highlights how crosstalk towards and from members of the NVU are routed in three distinct categories. The green arrows represent the magnitude and directionality for which factors involved in nutrient support (i.e. growth and pro-survival factors) are released. The blue arrows represent the release of maintenance solutes (such as glucose, amino acids, Na^+^, K+, Cl^−^, HCO3^−^, Ca^2+^, and other ions etc.,). The pink arrows signify immunomodulatory signals. The left-hand side is indicative of homeostatic conditions whereas the right-hand side shows neuroinflammation during neuroHIV.

Near the BBB, cells of the immune system and the endothelium are actively engaged in crosstalk (Figure 1). Infected immune cells release signaling molecules such as chemokines, chemoattractants, viral components and bioactive material in immune-generated EVs that signal to ECs. Immune cells release chemokines such chemokine ligand 2 (CCL2) [23], which promote the accumulation of extra immune cells nearby. ECs are stimulated by this process, which also increases endothelial expression of adhesion molecules to aid in further immune cell recruitment [24, 25]. Once monocytes enter the parenchyma they differentiate and have increased susceptibility to infection. HIV_Bal_ infected macrophages can secrete miRNA that impact Toll-like receptors (TLR) signaling [26]. Peripheral ECs ubiquitously express TLR2 and TLR4 [27], however, brain ECs appear to respond primarily to TLR3, TLR4 and TLR7 ligands [28] offering these signaling pathways as available mechanisms of interaction.

In addition to the release of cytokines, immune cells can release extracellular vesicles (EVs) that signal to brain ECs (Figure 1). Monocyte and infected T-cell derived EVs trigger EC upregulation of adhesion molecules [29, 30], reactive oxygen production [29] and generation of inflammatory cytokines [30] promoting a positive feedback loop to increase immune infiltration. The mechanism by which these EVs induce these responses are not fully elucidated. Moreover, blood plasma and derived EVs have been shown to contain HIV virotoxins such as, Nef [31–33], gp120 [34] and Tat [29]. These HIV viroproteins can directly damage the vasculature.

Tat concentrations in the sera of HIV+ individuals have been reported to be between 2 and 40 ng/mL [35] and between 0.2 and 6.5 ng/mL in CSF [36], however, local concentrations may be higher in tissues. The effects of Tat on brain ECs have been well studied, with Tat decreasing EC expression of tight junctions and increasing permeability, as a function of concentration [37–40]. Tat subtype may also impact the magnitude of the BBB damage [41]. These alterations in TJ expression occur through the Rho/Rock pathway, MLCK and increased matrix metalloproteinase-9 (MMP-9) expression/activity [37, 39, 40, 42] which has been observed in HIV-1 Tg animals [43]. Tat can also induce reactive oxygen species (ROS), and phosphorylation of vascular endothelial-cadherin (VE-Cadherin) and beta-catenin which destabilizes the TJ complex [44]. Interestingly, EVs from ART naïve and ART treated PLWH induced similar effects [45] which indicates that ART does not fully mitigate the effects on EV production or composition.

Similarly to Tat, Nef has been detected in the plasma of PLWH [32, 33]. Moreover, levels of Nef remained elevated in PLWH on ART with suppressed plasma HIV RNA levels and in elite controllers [32]. Nef blood concentrations ranged from 2 to 16 ng/mL [33] and nef was found contained in EVs that were CD45+ indicating an immune source [32]. This is supported by studies on EVs isolated from HIV infected lymphocytes (J1.1 HIV-1-infected T cells) which contained nef [46]. Nef exposure can have a direct effect on the BBB. When expressed by an HIV-1 based vector, nef induces apoptosis in brain ECs [47] and increases BBB permeability *in vivo* in an MMP-dependent manner [48].

gp120 is also found in the serum of HIV-infected patients [49], and is estimated to range from 120 and 960 ng/mL [50]. However, later studies found lower gp120 concentrations between 1 and 15 ng/mL, possibly due to examination of acute versus chronic infection [51]. Like nef, gp120 has been detected in EVs isolated from J1.1 HIV-1-infected T cells [46]. Regarding the effect of gp120, several studies have examined the effect on the BBB. Gp120 (HIV-1MN, HIV-1_CM235_, HIV-1_Bal_ and R5 gp120) exposure increases brain endothelial cytotoxicity, permeability and transmigration of monocytes [52, 53]. Direct injection of SV(gp120) (a recombinant Tag-deleted SV40-derived vector expressing HIV-1NL4-3 gp120) into the caudate-putamen of rats also caused BBB leakage which was accompanied by increased MMP activity, and the loss of tight junction expression (claudin-5) [54].

Overall, the literature is very clear that exposure to cytokines or HIV viroproteins released by infected cells can exert a deleterious effect on the BBB thus disrupting homeostasis of the NVU.

## Pericytes to endothelial crosstalk

Pericytes which encircle the abluminal endothelial surface are critical in the finely tuned maintenance of the BBB. Crosstalk between pericytes and ECs, occurs though the shared basement membrane (spanning 20 nm) [55] or gap junctions that connect these two cell types directly together [56]. In the brain, the ratio of pericyte to EC is highest at 1:1, due to the need for exquisite control over cerebral blood flow [57]. Pericyte-endothelial crosstalk has been extensively studied, illuminating several signaling pathways (Figure 1). Pericytes release factors including angiopoietins, sphingosine-1-phosphate (S1P) and notch signaling molecules that are involved in the regulation of permeability, proliferation, migration and differentiation [58].

In the context of HIV, several studies have shown that NeuroHIV leads to a reduction in pericyte vascular coverage [59–61]. Brains of PLWH had reduced expression of platelet-derived growth factor receptor PDGFR-β, and CD13, which was observed even in PLWH on ART [59]. The results of reduced PDGFR-β was also found in humanized mice infected with HIV and HIV-1 transgenic mice (Tg26) [62]. However, studies in SIV infected macaques have given mixed results, with one reporting hypertrophied pericytes and a reduction in PDGFR-β expression only in SIVE lesions [63] while another did not have a reduction in PDGFR-β expression [62]. It is unclear if these discrepancies are a result of divergences in SIV infection or the experimental conditions.

Changes in pericyte vascular coverage may be due to the impaired crosstalk between pericytes and the endothelium (Figure 1). Pericytes exposed to pro-inflammatory cytokines reduced the release of transforming growth factor-beta (TGFβ) and angiopoietin-1 (Ang-1) and increased mRNA expression of key inflammatory cytokines (monocyte chemoattractant protein-1/MCP-1, IP-10/CXCL10, Rantes/CCL5) [59] which can signal for enhanced immune infiltration. The reduction in TGFβ and Ang-1 would reduce barrier stabilization and encourage a more angiogenic BBB. Interestingly, in PLWH, increased plasma ANG-2 levels correlated with higher plasma HIV-1 RNA after ART initiation [64] and effective ART was associated with higher ANG-1 levels [65]. This may suggest a role of vasculature maintenance in regulating effective ART control of viremia. Pericytes have also been reported to release inflammatory cytokines (G-CSF, granulocyte-macrophage colony-stimulating factor (GM-CSF), IL-1α, IL-5, IL-6, IP-10, keratinocyte chemoattractant (KC), MCPI-1, RANTES) in response to lipopolysaccharide (LPS) which increases free HIV virus crossing the endothelium more than when pericytes were not present. The increased virus crossing was proposed to be due to changes in endothelial transcytosis due to the pericyte derived MCP-1 [66].

Aside from alteration in cytokine production, pericytes communicate to the endothelium through gap junctions and the release of EVs [67]. HIV related studies have shown that a small number of pericytes can become infected by HIV (X4-tropic _NL4-3_, R5-tropic JR-_CSF_ HIV_YU-2_, HIV_49.5_, and EcoHIV) [68, 69]. Pericyte infection with HIV altered gap junction expression and increased communication with brain ECs [69]. Additionally, infected pericytes had increased interleukin-6 (IL-6) production and would disrupt BBB integrity [70]. Pericytes, like nearly all cells, can produce EVs [67]. However, to date no studies have been published that specifically analyze pericyte EV populations or EV crosstalk in the context of HIV infection.

## Astrocyte to endothelial crosstalk

Crosstalk between astrocytes and the endothelium remains understudied. Astrocyte endfeet form the glia limitans which wrap around and interact in close proximity with cerebral blood vessels. Astrocytes communication with the endothelium can promote BBB maintenance or induce hyperpermeability and aid in the recruitment of leukocytes into the perivascular space. Astrocytes are known to regulate the BBB through the release of soluble factors (Figure 1) [71]. Experiments to understand the interactions between these two cell types have focused on co-culture experiments that rely on the polarized nature (basal vs. apical sides) of the ECs [72]. Examples of astrocyte secreted paracrine factors that are known to regulate endothelial biology include production of vascular endothelial growth factor A (VEGF-A) which induces an EC transition from barrier forming to angiogenic and disassembles the tight junction complex [73]. Astrocytes can also secrete cytokines which are known to increase BBB permeability (i.e., Tumor necrosis factor-alpha/TNFα, Interleukin-1 beta/IL-1β, TGFβ) [74–77].

In contrast, astrocytic production of sonic hedgehog (Shh) promotes increased endothelial tight junction protein expression [78] and the release of wingless/integrated (Wnt) growth factors that regulate endothelial caveolae expression [79], both of which help maintain BBB stability. Changes in Shh production as a result of HIV/SIV infection have resulted in seemingly opposite results, with one study indicating decreased production in a humanized mouse model (HIV pNLEG1-70) [80] and another found only increased Shh within endothelium of lesions in simian immunodeficiency virus (SIV) infected rhesus macaques [63].

Studies on astrocytes in the context of HIV infection have focused on the effect of the HIV protein, trans-activator of transcription (Tat). Tat (HIV_LAI_) exposure results in induction of PDGF-BB expression, increased proliferation and release of MCP-1 and IL-1β [81]. Production of MCP-1 and IL-1β could result in increased BBB permeability and signal to recruit immune cells into the CNS. Increased cytokine production has also been observed *in vivo* in tetracycline-inducible glial fibrillary acidic protein (GFAP)-driven HIV-1 Tat transgenic mice (rtTA-Tat) with induced Tat protein increasing cortical expression of CCL2, CCL5, chemokine (C-X-C motif) ligand-1/CXCL1, TNFα, IL-1β, IL-6, IL-10, IL-17 [82]. However, the source of these pro-inflammatory responses becomes admittedly more complex to decipher. In addition to the production of cytokines, a small number of astrocytes can become infected (∼4–8%, HIV_ADA_, HIV_NL4-3_ strain) [83, 84]. When co-cultured with brain ECs, HIV_ADA_ infected astrocytes increased BBB permeability and EC apoptosis [84]. Further examination of the astrocyte endfeet showed dysregulated astrocytic processes to the ECs [84] identifying a role for aberrant crosstalk in the heighted BBB dysfunction.

Astrocytes also regulate the supply of nutrients (glucose and oxygen) through the release of mediators that signal to cerebral microvessels to regulate blood flow. Increases in intracellular calcium in astrocytes leads to the release factors that cause vasodilation and vasoconstriction, including epozyeicosatrienoic acids (EETs), prostaglandin E2 (PGE2), nitric oxide, arachidonic acid (AA) and 20-hydroxyeicosatetraenoic acid (20-HETE) [85]. Tat and gp120 has been shown to increase astrocyte calcium and result in vasodilation of capillaries [86]. While short term stimuli to elicit calcium changes may aid in NVU homeostasis, prolonged exposure to HIV viroproteins likely contributes to a chronic reactive astrocyte phenotype and dysregulated cerebral blood flow (CBF).

Astrocytes can also release extracellular cellular vesicles which contain proteins, mRNA and miRNA which may regulate the BBB [87]. These EVs may signal directly to the endothelium or end up in circulation. Because of the cargo EVs can carry and the recent timeline in which EVs have been recognized, some earlier studies which have examined the effects of conditioned media may actually be studying the effects of EVs over soluble factors. Astrocyte EVs in normal culture conditions shed EVs containing VEGF and fibroblast growth factor-2 (FGF-2) [88]. Like VEGF, FGF-2 has been shown to promote endothelial cell proliferation and angiogenesis [89]. In the context of HIV, Tat protein has been shown to induce the release of EVs containing miRs [90, 91] and nef [92]. As discussed in an earlier section, Nef has been shown to induce apoptosis in brain ECs [47]. However, few studies have examined the effects of astrocyte EV directly on brain ECs.

Aside from soluble factors and EVs, astrocytes have been reported to communicate through tunneling nanotubes [93] in the context of HIV [94]. However, this form of communication has not yet been shown as a means of crosstalk with the endothelium.

## Microglial to endothelial crosstalk

Microglia are known to survey the brain and play an important role in the response to injury and infections. Microglia play an important role in vasculogenesis during development but depletion studies during adulthood show that loss of microglia does not impact BBB integrity [95]. A recent study showed that microglia migrate to blood vessels during inflammation, initially assisting in the maintenance of the BBB through the release of CCL5. However, during chronic inflammation, microglia release cytokines, phagocytose astrocyte endfeet and contribute to BBB dysfunction [96]. Moreover, microglia can amplify the effects of a mild inflammatory insult to disrupt barrier integrity [97], and media from quiescent microglia can inhibit brain EC proliferation through released cytokines (TNFα) [98]. Additionally, soluble factors released from microglia can differentially alter endothelial tight junction expression [99]. Microglia, similar to pericytes, can release notch signaling molecules that can activate endothelial angiogenic pathways [100].

Microglia are susceptible to HIV infection, and once infected will release ATP, arachidonate, excitatory amino acids (glutamate, quinolate, and cysteine). These substances increase the immunity response in the CNS and can lead to abnormal activity of the BBB [22]. Microglia exposed to HIV viroproteins become activated and release ROS, cytokines (TNFα, IL-1β, MCP-1), calcium and nitric oxide [22]. These signaling molecules can directly impact the BBB to increase permeability, or indirectly activating other cells of the NVU. Moreover, infected microglia (HIV-1_ADA_) increased monocyte transmigration across an endothelial/astrocyte model more than with uninfected microglia or infected monocyte-derived macrophages (MDMs); thus highlighting unique aspects of microglia infection by HIV [101].

Aside from release of cytokines and signaling molecules, microglia also produce EVs which can impact the BBB. Similar to immune cells, infected microglia can produce increased levels of EVs. These EVs contained Nef which reduced brain endothelial TJ expression, permeability, and integrity [102]. HIV viroproteins can also induce microglial EV production and these EVs were shown to contain NLR family pyrin domain containing 3/NLRP3 and IL-1β which could disrupt the BBB [103].

## Aberrant NVU crosstalk disrupts neuronal function

Communication between the neurons and vasculature is critical for homeostasis (Figure 1). Neuronal activity regulates angiogenesis, vasodilation, BBB integrity and expression of BBB efflux transporters. Examples of crosstalk include signaling between VEGFR2 and N-methyl-d-aspartate receptor (NMDAR), the VEGF/VEGFR2 pathway, and secretions of Semaphorin3G [104].

Despite the fact that HIV-1 cannot infect neurons directly, neuronal damage is a crucial component in the development of HAND [105]. The neurodegeneration and neurological symptoms in HAND can be explained by both direct and indirect mechanisms. Direct mechanisms include viral toxins, cytokines and inflammatory factors that alter neuronal integrity. Indirect actions include compromising the BBB, which reduces nutrient support, or maintenance to the neurons. Examples of direct mechanisms include neurotoxic host factors released from HIV-1 infected macrophages and microglia [105, 106]. Numerous soluble factors, including chemokines (CCL2, CCL5, CXCL10), platelet activating factor (PAF), nitric oxide (NO), matrix metalloproteases (MMP), arachidonic acid, and pro-inflammatory cytokines (TNFα, IL-1, IL-6, interferons/IFN, and IL-8), are also released by activated macrophages, microglia, and astrocytes that disrupt neuronal function causing neuronal injury [107]. In the HAND brain, the disruption of glutamate-glutamine equilibrium is a major cause of the “indirect” damage to neurons. The damage is also seen to extend to vulnerable neural stem cell niches. Released by infected cells, the actions of gp120 and Tat on neurons is mediated through NMDAR, low density Lipoprotein Receptor related Protein (LRP) and chemokine receptors (CCR5 and CXCR4) [108, 109]. Activation of NMDAR by HIV-Tat is known to promote calcium cytotoxicity [110].

Indirect mechanisms at the BBB are manifested as reduced pro-survival nutrient support for neurons and altered extracellular neurochemistry needed for effective synaptic transmission. Glucose and oxygen are delivered to the brain through changes in the blood flow and BBB transport. Multiple studies have shown that virally-suppressed PLWH experience measurable alteration of resting neurovascular uncoupling, which included blood flow alterations and impaired whole or regional brain cerebral vascular reactivity (CVR) [111–113]. Another study found that PLWH experience hypermetabolism, but this was reversed following ART treatment. Notably, after 2 years of uninterrupted ART, several brain regions had hypometabolism which indicated continued or unresolved damage [114]. The underlying mechanism for this neurovascular uncoupling in the context of HIV remains unknown.

HIV infection can also alter A-beta (Aβ) secretion and accumulation which impacts NVU crosstalk. Early histology showed increased Aβ deposition, formation of amyloid plaques and neurofibrillary tangle-like structures [115, 116]; However, recent studies with neuroimaging have found no differences in Aβ deposition in PLWH that were cognitively impaired or normal [117–119]. The lack of imaging differences may be due to a more diffuse plaque deposition which is supported by a study of tissue from the National NeuroHIV Tissue Consortium (NNTC) [120].

HIV or its viroproteins can potentially induce changes in Aβ clearance/production resulting in increased Aβ accumulation that impacts all the components of the NVU and the endothelium directly. Studies on the effect of HIV viral proteins (specifically Tat) have showed increased Aβ secretion in rat hippocampal cell cultures [121] and altered Aβ degradation/formation by changes in neprilsin enzyme function, amyloid precursor protein (APP) cleavage, and Aβ converting enzyme (BACE-1) activity [116]. These changes in the regulation of Aβ have been found in a humanized mouse model of HIV infection which had Increased Aβ deposition and BBB dysfunction [122]. The HIV virus has been shown to alter brain EC expression of the receptor advanced glycation end products (RAGE), increase cellular uptake of Aβ as well as transfer of Aβ across the monolayer [123]. Aβ is directly toxic to ECs directly causing increased permeability, impaired glucose transport, apoptosis, and ROS production [124]. Additionally, excessive Aβ promotes oxidative stress and in turn can alter endothelial clearance of Aβ [125]. The effects of neuroHIV on Aβ, highlight the interconnected aspects of the NVU which can disrupt multiple cellular crosstalk avenues.

## Effects of ART drugs on the NVU

Although the prevalence of HAND has significantly decreased with the advent of anti-retroviral therapy (ART), PLWH have been found to have rising levels of neurocognitive impairment in the post- ART era [126]. *In vivo* and *in vitro* investigations on the peripheral nervous system and peripheral neurons have demonstrated that some antiretrovirals (ARVs) are neurotoxic [127]. These ARV associated neurotoxicity is influenced by alterations in lipid and protein metabolism, mitochondrial damage, and oxidative stress [128]. Unexpected outcomes from the administration of ART include changes in signaling events across the BBB and inside the brain parenchyma, which may impair CNS function. These negative impacts could result from a variety of causes, such as: changes in endothelial cell P-glycoprotein (Pgp) expression and caveolar changes; direct anti-retroviral drug interactions with glial cells and neurons; toxic or inflammatory factors produced in response to ART-induced systemic complications, such as hyperlipidemia and hypersensitivity; and selective pressure for mutation, resistance, and compartmentalization of the virus [129].

Only a few case reports have examined the impact of ART on the BBB, where patients with HAND shown an initial improvement of endothelial cell function following the initiation of ART, although eventually dysfunction reappeared [130, 131]. Echeverria et al. also observed endothelial dysfunction after treatment with rilpivirine, an antiretroviral [132]. An *in vivo* study by Bertrand et al. demonstrated that ART can disrupt the BBB function and increase HIV associated cerebrovascular pathology [133]. Additionally, the combination of comorbidities, such as hyperglycemia may exacerbate ART toxicity [134].

ART may contribute to altered astrocyte-endothelial crosstalk. Although few studies have examined this concept, one study found that astrocytes exposed to ART drugs increase TGFβ3, IL-1α, IL-1β, and VEGF-A expression [135]. Secretion of these soluble factors could have an effect on endothelial function and increase BBB permeability or angiogenic responses. Treatment with these particular ART drugs did not increase MCP-1 expression [135] which was frequently seen with exposure to HIV proteins. Further studies to examine other ART drugs and the long-term consequences of ART are needed to understand whether these effects have acute changes or sustained alterations in Astrocyte-EC communication. An additional way in which ART may impact the NVU is through altering levels of Aβ. Specifically, protease inhibitors have been shown to inhibit insulin degrading enzyme which cleaves Aβ [136], which would potentially contribute to Aβ accumulation.

As indicated above, several cell types including ECs, pericytes, and astrocytes require synergy for the BBB to remain intact. Overall, the toxicity of ART directly or indirectly on various cell types must be taken into account given the significance of cell-cell contact in the NVU, since the toxicity observed on one cell type is likely to alter the BBB functioning.

## Xenobiotics of abuse further affects NVU crosstalk during HIV neuroinflammation

Xenobiotics of abuse are chemical substances that are foreign to the body and have addictive properties and/or induce dependence. Use of drugs of dependence are associated with increased risk of HIV infection [137] and can significantly alter the NVU independent from HIV infection [138]. However, drugs such as opioids [139], psychostimulants [140], and alcohol [141] can synergistically interact with pathologies induced by HIV infection to further impact immune function. Clinically, drug use in the context of HIV dramatically aggravate neurocognitive illness in HIV patients [142, 143], and thus drug-NVU interactions in the context of HIV may have important clinical effects impacting patient outcomes.

Drugs of dependence interact with brain circuitry to maintain use, including the mesolimbic brain reward pathway [144]. It is thought that drugs “hijack” the brain reward pathway [145] by driving specific neural circuits and cell types that then increase use motivation. Decades of research have uncovered a key role of the glutamatergic tripartite synapse [146] within the nucleus accumbens core (NAcore) [144, 147, 148], which is thought to encode the transition to substance use disorder from occasional use. Neurons within the NAcore receive a large number of presynaptic glutamatergic afferents from the prefrontal cortex (PFC) [149], basolateral amygdala (BLA) [150], ventral hippocampus (vHIPP) [151], midline/intralaminar thalamic nucleus [152], and glutamatergic neurons from the ventral tegmental area (VTA) [153]. GABAergic post-synaptic medium spiny neurons comprise the majority of cells within the NAcore and receive glutamatergic input from these cortical regions. However, astrocytes, which ensheathe synapses via perisynaptic astroglial processes (PAPs) and also communicate with the endothelium as described above, have come into focus as playing a critical role in driving drug seeking behavior [154]. Importantly, during withdrawal from various drugs including cocaine [155] and heroin [156], astroglial endfeet retract from synapses within the NAcore, and reinstated drug seeking behavior induces a rapid return of astroglial endfeet to synapses [156] as well as a rapid increase in expression of the glutamate transporter GLT1 [157]. Further, neuroimmune signaling plays a key role in driving drug seeking behavior [157]. Although the role of microglia in driving drug use motivation is still unclear, studies have shown that these immune cells are structurally and/or functionally changed following drug exposure including with nicotine [158], alcohol [159], and opioids [160]. Taken together, there is evidence that drugs of dependence significantly dysregulate neuroimmune and glutamatergic signaling, and specifically, reduce astroglial contact with synapses within the reward pathway. This may have important implications for HIV as astroglia may serve as viral reservoirs and may therefore play a role in the pathogenesis of brain HIV infection in the context of substance use [161]. Importantly, no studies to date have evaluated dysregulated glutamate homeostasis following volitional drug use in the context of HIV, reflecting a large gap in the field.

Although the role of neuroimmune signaling in addiction processes is still a topic of investigation, it is important to note that both drugs of dependence [138] and HIV disrupt the integrity of the BBB. Drugs including fentanyl [162], methamphetamine [163], mephedrone [164], morphine [37] and nicotine [165] severely disrupt the BBB and exacerbate immune responses. These drugs can also exacerbate the effects of HIV or HIV viroproteins on the BBB [37]. Importantly, drugs can have important implications for HIV replication in the CNS through directly increasing viral replication or altering ART CNS-penetration [166]. While many studies have examined the effects of drugs on the former, our understanding of changes in CNS ART concentrations is limited. Opioids have been shown to decrease brain concentrations of some antiretroviral drugs and also increase leakiness of tracers indicating increased breakdown of the BBB [167]. Although increased permeability of the BBB can lead to increased neurotoxicity, one study found that nicotine increased antiretroviral drug exposure in the brain [168], which reflects a potentially important drug-drug interaction at the BBB. Studies evaluating BBB integrity during HIV infection and changes in ART CNS penetration following volitional use of addictive drugs are needed to fully understand these interactions.

Use of drugs across drug classes (including opioids and psychostimulants) in HIV patients has meaningful impact on neuroimmune function. For example, one study showed that PLWH who also used opioids had greater brain microglial activation levels [169]. Another study revealed that opioids may play a significant role in HIV immunopathogenesis by enhancing the HIV R5 strain infection of macrophages by upregulation of CCR5 receptor expression and elevation of chemokine expression [170]. In another study, methamphetamine (METH) usage worsened neuronal dysfunction in brains of HIV-1 patients [171]. Specifically, patients with HIV encephalitis that used methamphetamine showed a higher frequency of ischemic events and increased microglia reactivity as compared to patients with HIV encephalitis that did not use methamphetamine. Cocaine also has been shown to speed up the pathophysiology associated with HAND. In one study, the authors suggest that enhanced monocyte adherence and transmigration into the CNS is mediated by cocaine through the activation of ALCAM on ECs [172].

Above we describe drugs of dependence which have been shown to exert deleterious effects on the NVU in the context of neuroHIV, cannabinoids (CB; including phytocannabinoids) seem to mediate some degree of neuroprotection in preclinical studies [173] and thus have been posited as a potential treatment avenue for PLWH. The positive effects of CBs on the NVU include protection of BBB integrity [174, 175] and increased proliferation and migration of endothelial cells [176] which have positive effects in models of disease states such as diabetes [177]. Thus, this family of compounds could have therapeutic potential in the context of HIV. Although there are deleterious health effects induced by chronic use of Δ9-tetrahydrocannabinol (THC) [178] including hyperemesis, mood disorders, neurocognitive impairment, peripheral vascular diseases, among others [179], there is mechanistic evidence that CBs have beneficial effects in the context of HIV. Lu and colleagues show that after being harmed by HIV-1 Gp120, human brain microvascular endothelial cells (HBMEC) and the BBB can be restored to their original integrity by treatment with cannabinoid agonists [180]. In another study, the synthetic cannabinoid agonist WIN55,212-2 protected against gp120-induced neuronal damage in human dopaminergic neurons, specifically blunting impairments in dopamine transporter function, apoptosis, and lipid peroxidation [109]. Further, these protective effects were mediated via the CB2 receptor as well as microglia. Interestingly, another study found neuroprotective effects of N-arachidonoylethanolamine (anandamide/AEA) and 2-arachidonoyl-glycerol (2-AG) mediated via the CB1 receptor [181]. Together, these preclinical studies implicate the endocannabinoid system in potentially having impactful neuroprotective effects and may be a viable pharmacotherapeutic avenue for medications development in HIV. However, there are clinical concerns for PLWH who also smoke marijuana given the increased risk for pulmonary disease [182] which may be enhanced by marijuana use [183]. Thus, drug development efforts may be needed to enhance potential CB therapeutics that do not have addictive properties but retain beneficial effects on the NVU.

## Perspectives, future directions, and conclusions

In the recent years, there has been a growth in our understanding of the precise host factors that mediate the neurotoxic insult brought on by exposure of the CNS to HIV-1-infected cells, HIV-1, or particular HIV-1 proteins. Researchers are learning more about the mechanisms that alter and impede NVU crosstalk. However, future progress will depend on improved ability to evaluate how NVU crosstalk is changed as a consequence of newly emerging HIV genetic variants that impact disease severity. For better understanding of these mechanisms, it would be beneficial to have improved translational models that replicate the crucial connections between cells and the dynamic nature of the NVU.

Many studies have been performed to study HIV infection and neuropathogenesis *in vivo* (Nonhuman primates, humanized mice, EcoHIV, and transgenic rodent models are a few of them). These models remain essential for our study of NIH neuroinvasion and pathogenesis. The continued refinement of the animal models for HIV will provide further understanding of how NVU crosstalk becomes compromised, how it compensates, whether damage is repairable as a function CNS viral reservoirs, antiretroviral therapy, immune modulatory drugs or xenobiotics of abuse. Importantly, improved models (*in vitro* and *in vivo*) provide a practical platform to evaluate eradication strategies. Theirs is also a need to address combination of factors since current models are typically restricted to one or more particular aspects of clinical disease [184].

Organoids are one *in vitro* model that is currently being used to study HIV infection of the CNS [185–187]. Organoids are 3D miniatures of the organ that are made using stem cell progenitors and are being widely used explore neuroinflammations and neurodegeneration [188]. Unfortunately, organoids are currently limited without a functional vasculature. Recently, there have been studies that look at vascularizing an organoid through *in vivo* implantation [189], generating organoids which upregulated vessel forming transcription factors [190] or creating vessel organoids that are fused with brain organoids [191]. However, continuous perfusion of organoid vasculature, a critical aspect for the study of immune interaction and infiltration, remains a challenge.

We posit that the latest discoveries in material science and tissue engineering will enable us to create biomimetic models of the NVU that would allow for exquisite interrogation of NVU dynamics and biological processes in both health and disease. Spatially and cytoarchitectural relevant 3D models have the potential to accurately mimic this unique microenvironment. In a previous manuscript, we discussed multiple 3D models to study the NVU [192]. We also recently published a tissue engineered 3D model of the NVU which enables recapitulation of key physiological parameters using light-based bioprinting technology that helps bridge the transability gap for the development of new neuroprotective therapeutic strategies [8]. Future work that bridges key physiological aspects of organoids with perfusable vasculature systems may combine the critical elements of these types of models for more realistic and translatable discoveries in the field of NeuroHIV.

Overall, a continued improvement in the understanding of NVU crosstalk pathways and signaling factors, offers significant breakthroughs in better diagnosis, predictors of prognosis and therapeutic intervention that would lead to protection and restoration of neurological function in the face of neuroinflammation.
